# Lysine methyltransferase Kmt2d regulates naive CD8^+^ T cell activation-induced survival

**DOI:** 10.3389/fimmu.2022.1095140

**Published:** 2023-01-19

**Authors:** Jaekwan Kim, Thomas Nguyen, Jeffrey Cifello, Raheel Ahmad, Yongqing Zhang, Qian Yang, Ji-Eun Lee, Xiang Li, Yan Kai, Supriyo De, Weiqun Peng, Kai Ge, Nan-ping Weng

**Affiliations:** ^1^ Laboratory of Molecular Biology and Immunology, National Institute on Aging, National Institutes of Health, Baltimore, MD, United States; ^2^ Laboratory of Genetics and Genomics, National Institute on Aging, National Institutes of Health, Baltimore, MD, United States; ^3^ Laboratory of Endocrinology and Receptor Biology, National Institute of Diabetes, Digestive and Kidney Diseases, National Institutes of Health, Bethesda, MD, United States; ^4^ Department of Physics, George Washington University, Washington DC, WA, United States

**Keywords:** KMT2D, H3K4me1, naive CD8+ T cells, cell death, proliferation

## Abstract

Lysine specific methyltransferase 2D (Kmt2d) catalyzes the mono-methylation of histone 3 lysine 4 (H3K4me1) and plays a critical role in regulatory T cell generation *via* modulating Foxp3 gene expression. Here we report a role of Kmt2d in naïve CD8^+^ T cell generation and survival. In the absence of *Kmt2d*, the number of CD8^+^ T cells, particularly naïve CD8^+^ T cells (CD62L^hi^/CD44^lo^), in spleen was greatly decreased and *in vitro* activation-related death significantly increased from *Kmt2d* ^fl/fl^CD4cre^+^ (KO) compared to *Kmt2d* ^fl/fl^CD4cre^-^ (WT) mice. Furthermore, analyses by ChIPseq, RNAseq, and scRNAseq showed reduced H3K4me1 levels in enhancers and reduced expression of apoptosis-related genes in activated naïve CD8^+^ T cells in the absence of Kmt2d. Finally, we confirmed the activation-induced death of antigen-specific naïve CD8^+^ T cells *in vivo* in Kmt2d KO mice upon challenge with *Listeria monocytogenes* infection. These findings reveal that Kmt2d regulates activation-induced naïve CD8^+^ T cell survival *via* modulating H3K4me1 levels in enhancer regions of apoptosis and immune function-related genes.

## Introduction

Histone methylation plays an essential role in establishing cell-type specific gene expression and T cell differentiation (1–3). Studies of various types of histone methylation, including H3K4me3, H3K9me3 and H3K27me3, revealed diverse and contrasting roles in chromatin structure and in transcriptional regulation of T cells (4–6). Histone 3 lysine 4 mono-methylation (H3K4me1) is present in enhancer regions (7, 8) and is regulated by the SET-domain containing histone methyltransferases, Kmt2c and Kmt2d (9, 10), and histone demethylases (11, 12). H3K4me1 displays distinct patterns between regulatory T cells (Treg) and conventional T cells (Tconv) (13) and T cell specific knockout of *Kmt2d* results in compromised development of regulatory T cells *via* dysregulation of Foxp3 gene expression (14). The role of Kmt2d in CD8^+^ T cells has not been examined.

CD8^+^ T cells are key components of adaptive immunity to control both external pathogens and internal cancerous cells (15, 16). Upon engagement of antigen peptide presented by the class I MHC on the antigen-presenting cells, naïve CD8^+^ T cells undergo extensive cell divisions and differentiate to become effector and memory T cells (17, 18). After peak clonal expansion and subsequent pathogen clearance, antigen-specific effector CD8^+^ T cells undergo extensive contraction (19) *via* apoptosis (20–22). Apoptosis is induced through the intrinsic and the extrinsic pathways (23). The intrinsic pathway includes permeabilizing mitochondria and releasing cytochrome c into cytoplasm through caspase 9 whereas the extrinsic pathway is activated by the binding of death ligands to death receptors such as FasL to Fas, and results in the formation of the apoptotic signaling complex activating caspase 8 (20, 21, 24). T cell survival and death during an immune response are regulated by both intrinsic (expression of co-stimulatory and inhibitory receptors, telomere length) and external (nature of antigens, ligands for co-stimulatory and inhibitory receptors) factors in a highly controlled manner (3, 25, 26). Despite tremendous progress in understanding the core pathways and components, transcriptional control of activation and death regulators *via* chromatin structure has just begun to be understood.

Recent studies of histone methyltransferases reveal their roles in regulation of cell survival, death, and diseases (27, 28). Loss of Setdb1, one of the major histone methyltransferases, results in histone 3 lysine 9 trimethylation (H3K9me3), results in impaired CD8^+^ T cell development in thymus (29). This impairment is partially due to the aberrant expression of FcγRIIB and increase apoptosis in single CD4^+^ or single CD8^+^ thymocytes (29). Ezh2 is a key histone methyltransferase that catalyzes histone 3 lysine 27 trimethylation (H3K27me3). Loss of Ezh2 leads to prolonged cell divisions and increases apoptosis in CD8^+^ T cells (30). Ezh2 regulates the repression of the key cell cycle regulators, Cdkn2a and Cdkn1c. Activation of CD8 T cells in the absence of Ezh2 leads to impairment of cell division and increased apoptosis (30). Kmt2d is a histone methyltransferase that modulates active chromatin methylations (31). Furthermore, knockdown of KMT2D suppressed cell proliferation and induced apoptosis in a gastric cancer cell line (32). However, it is unknown whether Kmt2d regulates CD8^+^ T cell proliferation and survival.

In this study, we investigated the role of Kmt2d in CD8^+^ T cell activation and apoptosis using T-cell specific Kmt2d knockout model. We found that Kmt2d knockout mice had a reduced number of peripheral naïve CD8^+^ T cells and a significant increase in apoptosis of naïve CD8^+^ T-cells post activation *in vitro* and *in vivo*. We showed that *Kmt2d* depletion led to reduced H3K4me1 in promoters and enhancers correlating with reduced mRNA levels of several apoptosis related genes, providing evidence of Kmt2d in regulation of activation-induced apoptosis of naïve CD8^+^ T cells. Finally, ChIPseq revealed that Kmt2d directly binds to promoters and enhancers of targeted genes. Collectively, these findings showed Kmt2d plays a critical role in the regulation of naïve CD8^+^ T cell generation and activation.

## Results

### Reduced circulating naïve CD8^+^ T cells in Kmt2d KO mice

**Figure 1:**
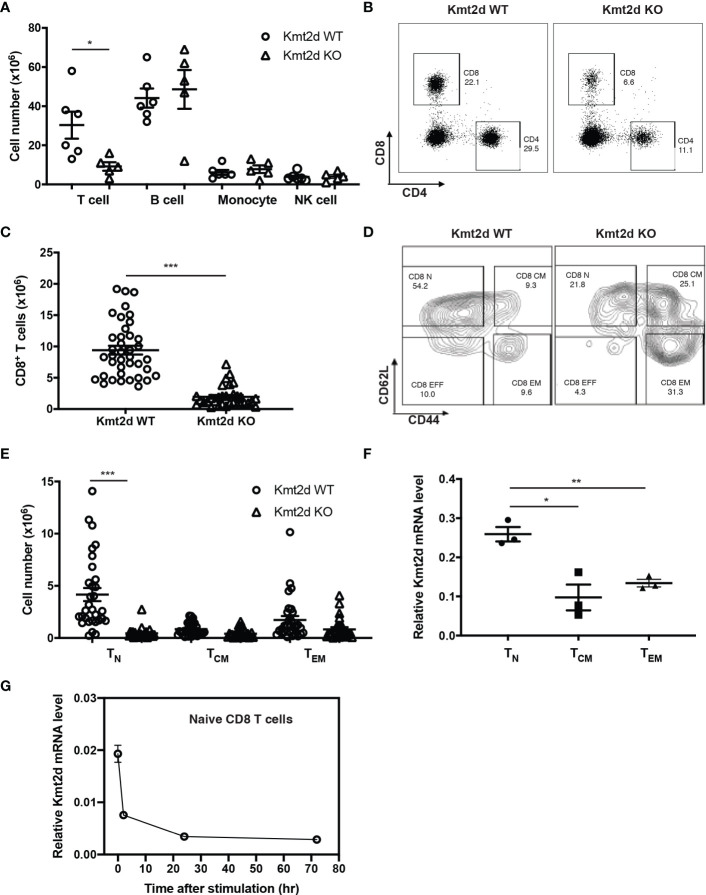
CD8^+^ T cell number is reduced in Kmt2d KO mice. **(A)** Immune cells in spleen (N=6, Kmt2d WT; N=5, Kmt2d KO). **(B)** Representative flow graphs of CD4^+^ and CD8^+^ T cells in spleen of Kmt2d WT and Kmt2d KO mice. **(C)** Number of CD8^+^ T cells in spleen of Kmt2d WT (N=38) and Kmt2d KO mice (N=27). **(D, E)** Number of CD8^+^ T cell subsets: Naïve (T_N_, CD62L^hi^/CD44^lo^), Central memory (T_CM_, CD62L^hi^/CD44^hi^), and effector memory (T_EM_, CD62L^lo^/CD44^hi^) in spleen of Kmt2d WT (N=38) and Kmt2d KO mice (N=27). **(F, G)** Relative Kmt2d mRNA level in CD8^+^ T cell subsets (N=3) and stimulated CD8^+^ T_N_ cells (N=3 each timepoint). Paired comparison was calculated by Student’s t-test; *p < 0.05, **p < 0.01 and ***p < 0.001.

To investigate the role of Kmt2d in CD8^+^ T cell function, we generated T-cell specific Kmt2d knock out mice (Kmt2d^fl/fl^-CD4cre^+^ or KO). Kmt2d-floxed mice (Kmt2d^fl/fl^) were crossed with CD4-Cre mice (9, 33), resulting in deletion of exon 16-19 of Kmt2d in all T cells (**Supplementary Figure 1A**). Kmt2d KO mice had no obvious difference in body weight and spleen size compared to the Kmt2d WT littermates but the numbers of splenocytes were significantly reduced (**Supplementary Figure 1B**), due to a decrease in both CD4^+^ and CD8^+^ T cells (**Figures 1A–C**). Within CD8^+^ T cells, naïve CD8^+^ T cells were reduced most whereas memory subsets were slightly reduced compared to the Kmt2d WT mice (**Figures 1D–E**). The difference in loss of CD8^+^ T cell subsets correlated with the mRNA level of Kmt2d expression in these subsets (**Figure 1E**). *In vitro* stimulation (anti-CD3 and anti-CD28 antibody or α-CD3/CD28) reduced Kmt2d expression in WT cells (**Figures 1F, G**). Further analysis of thymocytes showed maturation of CD4^-^CD8^+^ thymocytes were significantly impaired (**Supplementary Figure 2**). These findings suggest that Kmt2d plays a critical role in the generation of naïve CD8^+^ T cells.

### Increased cell death of naïve CD8^+^ T cells from Kmt2d KO mice in response to *in vitro* stimulation

**Figure 2:**
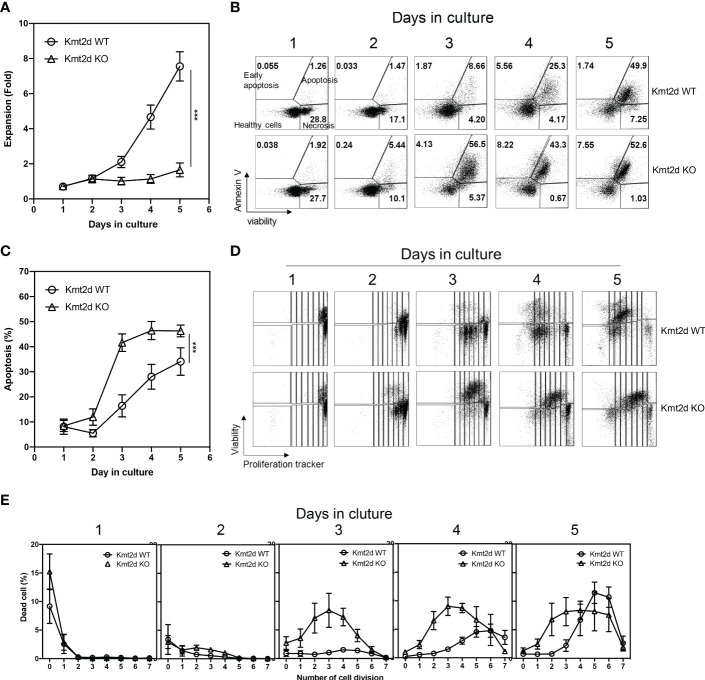
Kmt2d KO naïve CD8^+^ T cells have increased apoptosis upon stimulation driven proliferation. FACS-sorted naïve CD8**^+^** T cells from spleen were labeled with proliferation tracking marker and stimulated with immobilized anti-CD3 (3 μg/ml) and soluble anti-CD28 (1 μg/ml) antibodies up to five days. Three batches of independent experiments were performed with pooled naïve CD8**^+^** T cells from 3-6 mice (N=9 for Kmt2d WT, 6 for Kmt2d KO). **(A)** Cell number increase during 5 days of culture. Fold increase per seeded cell number was presented. **(B)** Representative plot of apoptosis analysis. **(C)** Average apoptotic cell ratio. **(D)** Representative display of proliferation tracker (efluor450) dilution and Ghost viability dye. **(E)** Summary of the dead cells ratio found on each division defined by efluor450 dilution and Ghost viability dye. ***, p<0.001 based on two-way Anova test.

To investigate the function of Kmt2d, we isolated naïve CD8^+^ T cells from Kmt2d KO and Kmt2d WT mice and analyzed their response to *in vitro* α-CD3/CD28 stimulation. We found a significantly reduced expansion of CD8^+^ T cells from Kmt2d KO compared to those from Kmt2d WT mice during the 5-day stimulation (**Figure 2A**). We stained naïve CD8^+^ T cells with Annexin V, a viability dye, and a tracking dye eFluor 450 (CPD-450) both prior to and post stimulation to determine whether the reduced expansion of naïve CD8^+^ T cells were due to increased cell death or reduced proliferation/cell division. We found a significant increase of apoptosis in stimulated naïve CD8^+^ T cells (day 3-5 after stimulation) from Kmt2d KO compared to Kmt2d WT mice (**Figures 2B, C**). But activation induced a similar number of cell divisions between Kmt2d KO and Kmt2d WT mice (**Figures 2D, E**). Intriguingly, cell death of naïve CD8^+^ T cells from Kmt2d KO mice was detected at first division but the death of naïve CD8^+^ T cells from Kmt2d WT mice was observed only at the 3^rd^ cell divisions (**Figures 2D, E**). These results demonstrated that Kmt2d regulates activation-induced apoptosis but not cell division in naïve CD8^+^ T cells.

### Increased expression and activity of apoptosis mediators in CD8^+^ T cells from Kmt2d KO mice

**Figure 3:**
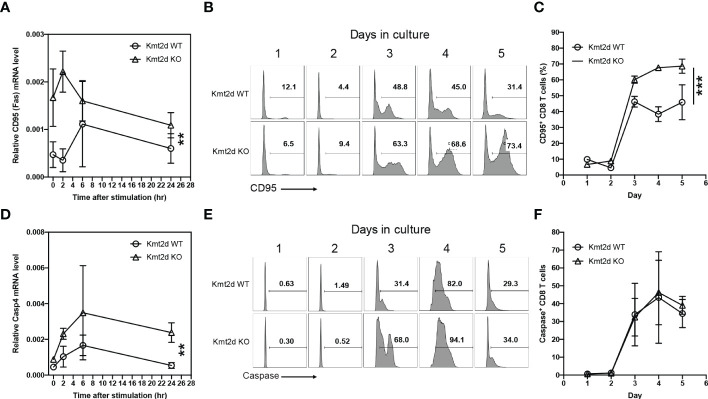
Increased apoptotic genes expression and caspase activated naïve CD8+ T cells of Kmt2d KO mice. **(A)** mRNA expression of Cd95 in naïve CD8+ T cells from Kmt2d WT and KO mice post one day stimulation with aCD3/CD28. **(B)** Representative histogram of CD95+ naïve CD8+ T cells after aCD3/CD28 stimulation. **(C)** Average percentage of CD95+ naïve CD8+ T cells after aCD3/CD28 stimulation from Kmt2d WT and KO mice (N=3). **(D)** mRNA expression of Casp4 in naïve CD8+ T cells post aCD3/CD28 stimulation. **(E)** Representative histogram of caspase activity at indicated time points. DEVDase activity was assessed using DEVD substrate-based assay. **(F)** Average of the caspase activity positive cells in percentage of total stimulated naïve CD8+ T cells (N=3). *p < 0.05; **p < 0.01 based on two-way Anova test.

To determine the mechanisms of increased apoptosis, we analyzed key cell death related genes (CD95 or Fas and Caspase activity). We found the expression levels of CD95 protein on the surface of naïve CD8^+^ T cells were significantly higher between 3 to 5 days after stimulation in Kmt2d KO than in Kmt2d WT mice (**Figures 3A–C**). We then measured caspase 3/7 activity and found a significant increase of activity in stimulated naïve CD8^+^ T cells from Kmt2d KO *versus* Kmt2d WT mice (**Figures 3D–F**). Together, increases of CD95 expression and caspase activity explain increased apoptosis of naïve CD8^+^ T cells from Kmt2d KO mice after *in vitro* stimulation.

### Reduced histone H3K4me1 in enhancer regions and reduced TNF expression in activated naïve CD8^+^ T cells of Kmt2d KO mice

**Figure 4:**
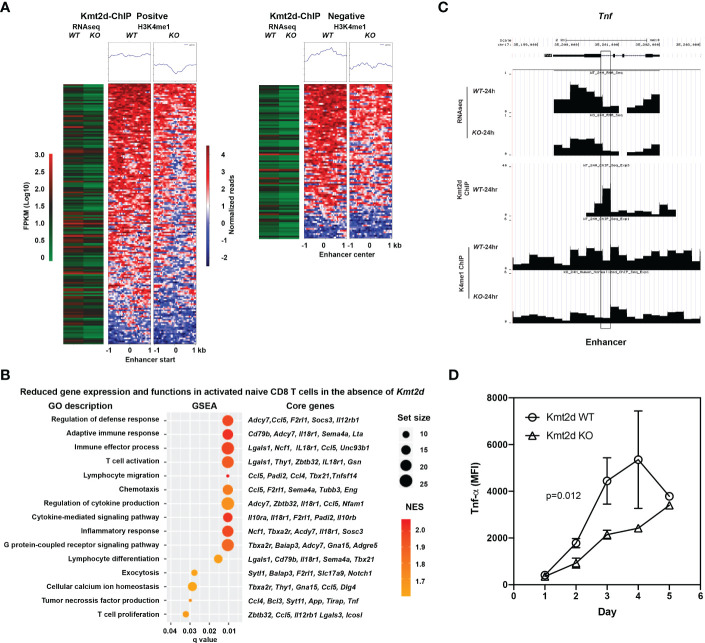
Reduced levels of H3K4me1 in enhancer regions and corresponding gene expression in activated naïve CD8^+^ T cells of Kmt2d KO mice. **(A)** Reduced gene expression associated with reduced H3K4me1 level in enhancer in Kmt2d KO activated naïve CD8^+^ T cells after stimulation. Naïve CD8^+^ T cells from spleen of both Kmt2d WT and Kmt2d KO mice by FACS-sort and stimulated *in vitro* by αCD3/CD28 antibody for 24 hours. Stimulated cells were harvested for RNAseq, ChIPseq of H3K4me1 and Kmt2d. Number of reduced expressed genes in Kmt2d KO mice along with H3K4me1 level and with (N=167) or without (N=90) Kmt2d binding are presented. **(B)** Selected reduced functions based on gene expression changes in activated naïve CD8^+^ T cells between Kmt2d WT and Kmt2d KO mice. Gene set enrichment analysis (GSEA) was used and significantly reduced GO functional groups (q<0.05) and their core changed genes along with gene set size, normalized enrichment score (NES), and q values are presented. **(C)** Expression (RNAseq), H3K4me1 level, and Kmt2d binding on *Tnf* gene. Gene track and ChIP-seq reads are presented and enhancer is marked. **(D)** TNF-α expression in stimulated naïve CD8^+^ T cells from Kmt2d WT and Kmt2d KO mice. TNF-α expression in stimulated naïve CD8^+^ T cells were determined by intracellular staining and flow cytometry. The median fluorescent intensity (MFI) was presented at each indicated time (N=3).

To determine the effects of the absence of Kmt2d on H3K4me1 and gene expression, we performed H3K4me1 ChIPseq, Kmt2d ChIP-seq, and RNAseq analyses in *in vitro* stimulated (anti-CD3/CD28) naive CD8^+^ T cells from Kmt2d KO and Kmt2d WT mice. As H3K4me1 is enriched in enhancers (34), we examined whether there is a reduction of H3K4me1 in enhancers in the absence of Kmt2d resulting in reduction of the corresponding gene expression. In the absence of Kmt2d, the overall levels of H3K4me1 in naïve CD8^+^ T cells may be reduced from those of Kmt2d WT mice but the PCR amplification during ChIP library preparation can diminish such difference. This may contribute to underestimation of H3K4me1 differences between the two genotypes. To precisely assess the H3K4me1 level, we spiked-in lysate of human naïve CD8^+^ T cells (10% of total) as an internal control during the ChIP step prior to PCR amplification. As the anti-H3K4me1 antibody recognizes H3K4me1 from both human and mouse cells, we were able to use the amount of human ChIP-DNA to normalize the difference of total H3K4me1 between Kmt2d WT and Kmt2d KO mice. We required differences of at least 1.6-fold in our analysis, to both H3K4me1 counts at the enhancer and the level of the corresponding gene expression, resulting in 257 altered genes (**Figure 4A**, **Supplementary Table 1**). We also confirmed the gene expression changes by quantitative RT-PCR of selected genes related to apoptosis (**Supplementary Figure 3**). To determine if Kmt2d directly regulates H3K4me1, we carried out Kmt2d ChIP-seq and found that Kmt2d was bound to approximately 65% (167 out of 257) of the enhancers with reduced H3K4me1 levels (**Figure 4A**). Further,we applied gene set enrichment analysis and identified several T cell functions were reduced including T cell activation, proliferation, migration, and cytokine-mediated signaling pathway in naïve CD8^+^ T cells from Kmt2d KO mice (**Figure 4B**). A key cytokine in T cell activation, tumor necrosis factor (TNF) was reduced at the mRNA level (RNAseq) and at the H3K4me1 enhancer level (35) in stimulated CD8^+^ T cells from Kmt2d KO mice (**Figure 4C**). Finally, we measured the protein level of TNF-α by intracellular staining and confirmed that TNF-α protein was significantly reduced in activated naïve CD8^+^ T cells from Kmt2d KO mice compared to those from Kmt2d WT mice (**Figure 4D**). Together, Kmt2d depletion resulted in decrease of H3K4me1 at enhancers and consequently reduced gene expression affecting a broad range of T cell functions including TNF-α.

### Altered expression of apoptosis-related genes in resting and stimulated individual naïve CD8^+^ T cells of Kmt2d KO mice

**Figure 5:**
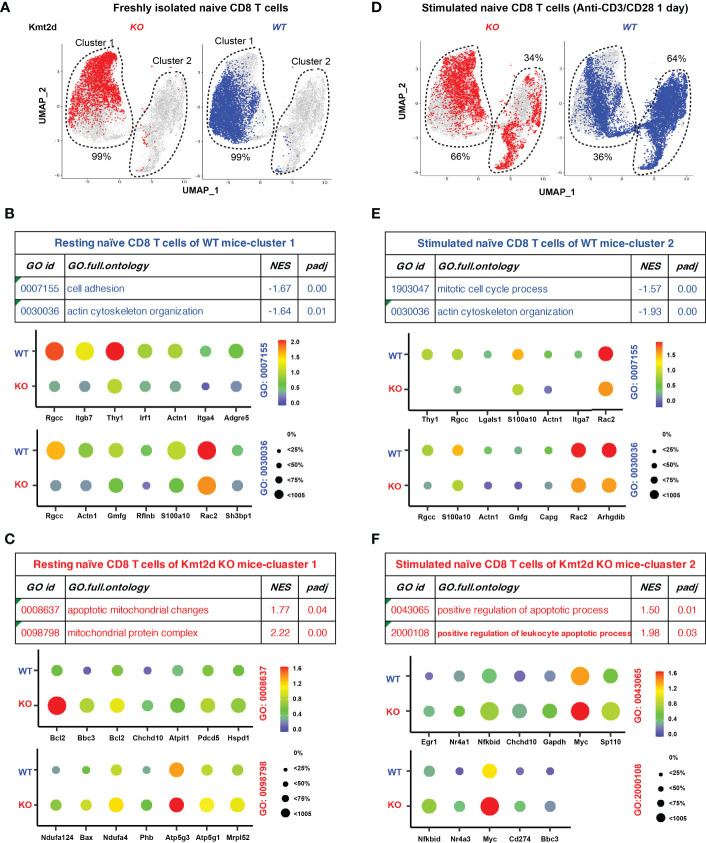
Altered expression of apoptosis related genes in single CD8^+^ T cells of Kmt2d KO mice. **(A)** UMAP of freshly isolated naive CD8^+^ T cells from Kmt2d WT and Kmt2d KO mice. **(B)** Enriched gene expression and associated GO functional groups analyzed by GSEA in freshly isolated naïve CD8^+^ T cells from Kmt2d WT mice. *NES* refers to normalized enrichment score. *padj* refers to adjusted p-value (FDR). **(C)** Enriched gene expression and associated GO functional groups analyzed by GSEA in freshly isolated naïve CD8^+^ T cells from Kmt2d KO mice. **(D)** UMAP of *in vitro* stimulated (antiCD3/anti-CD28 for 1 day). Percentages indicate of cells in each of two clusters. **(E)** Enriched gene expression and associated GO functional groups analyzed by GSEA in stimulated naïve CD8^+^ T cells from Kmt2d WT mice. **(F)** Enriched gene expression and associated GO functional groups analyzed by GSEA in stimulated naïve CD8^+^ T cells from Kmt2d KO mice.

Because naïve CD8^+^ T cells are heterogenous cells, we used scRNAseq method to compare transcriptome changes in individual resting and stimulated naïve CD8^+^ T cells from Kmt2d KO mice. Analysis of freshly isolated naïve CD8^+^ T cells showed that Kmt2d KO and Kmt2d WT mice had a similar overall gene expression pattern (Cluster 1 in **Figure 5A**). Further analyses of differential gene expression (DGE) and GSEA showed that naïve CD8^+^ T cells from Kmt2d WT mice had enriched gene functions in cell adhesion and actin cytoskeleton organization (**Figure 5B**) whereas naïve CD8^+^ T cells from Kmt2d KO mice were enriched in apoptotic mitochondrial changes (**Figure 5C**). After *in vitro* stimulation with anti-CD3 and CD28, stimulated naïve CD8^+^ T cells displayed an altered transcriptome (cluster 2 in **Figure 5D**) and the difference between Kmt2d KO and Kmt2d WT mice became more obvious both quantitatively (Cluster 2 accounted for 34% and 64% of cells, respectively) and qualitatively (**Figures 5E, F**). Further analyses of DGE and GSEA showed that stimulated naïve CD8^+^ T cells (cluster 2) from Kmt2d WT mice had higher gene expression in mitotic cell cycle process as well as cytoskeleton organization (**Figure 5E**) whereas stimulated naïve CD8^+^ T cells (cluster 2) from Kmt2d KO had increased gene expression in apoptosis (**Figure 5F**). This single cell transcriptome analysis showed both quantitative differences in activation of naïve CD8^+^ T cells and qualitative differences in the level of gene expression of both freshly isolated and activated naïve CD8^+^ T cells between Kmt2d WT and KO mice and suggested that Kmt2d regulates expression of genes involved in survival of activated naïve CD8^+^ T cells.

### Reduced antigen-specific CD8^+^ T cells in response to Listeria infection in Kmt2d KO mice

**Figure 6:**
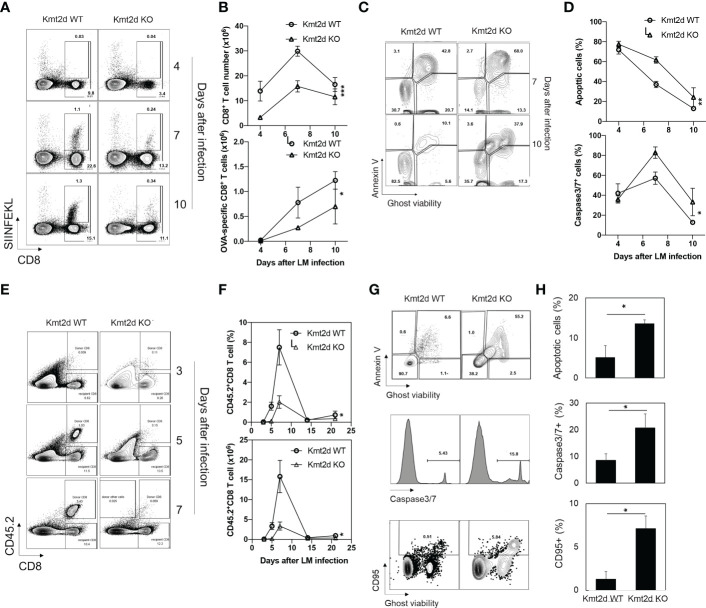
Reduced response of antigen-specific CD8^+^ T cells of Kmt2d KO mice to *Listeria* infection. **(A)** Representative plot of OVA-specific CD8^+^ T cells after LM-OVA infection. Kmt2d WT or Kmt2d KO mice were infected with 5x10^4^ cfu of LM-OVA and followed for 10 days. Number of OVA-specific CD8^+^ T cells from spleen were analyzed at indicated days post infection. SIINFEKL (OVA_257-264_)-dextramer was used for detection of OVA-specific CD8^+^ T cells. (N=3-4 each time point). **(B)** Apoptosis was analyzed using annexin V and Ghost viability dye at indicated days. **(C)** Representative plot of apoptosis analysis of OVA-specific CD8^+^ T cells in LM-OVA infected Kmt2d WT or Kmt2d KO mice at indicated days. **(D)** AnnexinV^+^/Ghost viability dye^+^ apoptotic cell (up) and Caspase3/7 activity positive cell (down) ratio in antigen specific cells (CD8^+^, SIINFEKL^+^, N=3-4 each time point). **(E-H)** Naïve CD8^+^ T cells from Kmt2d WT-OT-1 or Kmt2d KO-OT-1 mice were transferred to congenic mice and infected with LM-OVA. As OT-1 mouse has only OVA-specific CD8^+^ T cells, transferred cells were identified with CD45.2 and CD8. N=3-4 each day. **(E)** Representative plot of donor cells in congenic mice after LM-OVA infection at indicated time points. **(F)** Donor naïve CD8^+^ OT-1 T cells after LM-OVA infection. LM-OVA was injected 1day after donor cell transfer. **(G)** Representative plot of apoptosis analysis. AnnexinV/Ghost viability dye (top), active caspase3/7 (middle), and CD95 (bottom) expression in donor cells. **(H)** Average percentage of apoptotic cells, caspase active cells, and CD95^+^ cells in donor cells. The time course differences were calculated by two-way Anova test *, p<0.05, **, p<0.01 and ***, p<0.001. Paired comparison was calculated by Student’s t-test. *, p<0.05.

To determine whether the defects observed in naïve CD8^+^ T cells from Kmt2d KO mice *in vitro* occur *in vivo*, we applied the *Listeria monocytogenes*-OVA (rLM-OVA) infection model (36) in Kmt2d KO and Kmt2d WT mice. We injected 5x10^4^ CFU of LM-OVA *via* the tail vein to each mouse and OVA-specific CD8^+^ T cells were identified with the MHC-class-I-SIINFEKL dextramer (Immudex) in blood and spleen. OVA-specific CD8^+^ T cells were lower than 0.1% prior to rLM-OVA infection in both Kmt2d KO and Kmt2d WT mice. After rLM-OVA infection, total and OVA-specific CD8^+^ T cells were increased but the number of cells were significantly lower in Kmt2d KO than in Kmt2d WT mice (**Figures 6A, B**). Furthermore, we observed a significant increase of apoptosis (measured by both percentages of Annexin V^+^ and caspase-3/7 activity) of OVA-specific CD8^+^ T cells from Kmt2d KO mice compared to those from Kmt2d WT mice (**Figures 6C, D**). As reduced total naïve CD8^+^ T cell numbers (**Figures 1C, D**) could be a factor of the reduction of OVA-specific CD8^+^ T cells in Kmt2d KO mice post rLM-OVA infection, we adoptively transferred the same number (0.2 million per recipient mouse) of OVA-specific naïve CD8^+^ T cells isolated from Kmt2d KO-OT-I or Kmt2d WT-OT-I mice into congenic mice (CD45.1^+^) followed by rLM-OVA infection. Again, we observed significantly fewer OVA-specific CD8^+^ T cells from Kmt2d KO-OT-I than from Kmt2d WT-OT-I mice (**Figures 6E, F**). We also found significantly increased apoptosis as measured by percentage of Annexin V^+^, capase3/7 activity, and CD95 expression in OVA-specific CD8^+^ T cells from Kmt2d KO-OT-I compared to those from Kmt2d WT-OT-I mice (**Figures 6G, H**). Together, these results demonstrate that Kmt2d regulates activation-induced CD8^+^ T cell survival and death *in vivo* during *Listeria* infection.

## Discussion

Program cell death or apoptosis is an essential step post naïve CD8^+^ T cell activation and differentiation. The pathways of apoptosis are well characterized, and here we show that histone mono-methylation (H3K4me1) catalyzed by Kmt2d plays a critical role in survival during activation of naïve CD8^+^ T cells using a T-cell specific Kmt2d deletion mouse model. We showed that activation of naïve CD8^+^ T cells leads to a significant increase of apoptosis in the absence of Kmt2d under both *in vitro* and *in vivo* situations. The increased activation-induced apoptosis is due to increased expression of apoptosis-related genes without apparent defects in cell cycle by both bulk RNAseq and scRNAseq methods. In the absence of Kmt2d, the levels of H3k4me1 at enhancers and their regulating genes are substantially reduced, and consequently affect a broad range of functions of CD8^+^ T cells in response to activation. Collectively, our findings demonstrate that Kmt2d regulates naïve CD8^+^ T cell survival and activation-induced cell death and suggest that H3K4me1 catalyzed by Kmt2d plays a critical role in naïve CD8^+^ T cell function.

Activation-induced apoptosis is an essential process during T cell immune response and the timing of apoptosis is tightly regulated after T cell activation to achieve a balance between generation of sufficient effector cells to control an infection and removal of excessive effector cells after the clearance of the pathogen. *In vitro*, we observed that activation-induced apoptosis of naïve CD8^+^ T cells in the absence of Kmt2d occurs at the first cell division rather than 3^rd^ division in the presence of Kmt2d. This hasty activation-induced apoptosis significantly reduced expansion of antigen-specific effector CD8^+^ T cells in Kmt2d KO mice. Analyses of histone methylation and the transcriptome of activated naïve CD8^+^ T cells have revealed altered expression of a broad range of genes from Kmt2d KO compared to the Kmt2d WT mice, providing evidence that Kmt2d is critically important in regulation of apoptosis and immune functions of naïve CD8^+^ T cells. At the bulk cell level, not all enhancers have reduced H3K4me1 levels that have detectable Kmt2d binding and not all altered expressed genes have reduced H3K4me1 level at their enhancers. This demonstrates that regulation of H3K4me1 level and gene expression is complex and Kmt2d may preferentially regulate selected genes as reported (13). It is currently unknown how other methyltransferases such as Kmt2c function in the absence of Kmt2d (37). More studies will be needed to better understand the role of Kmt2d in CD8^+^ T cell activation and function.

Single cell transcriptome analysis reveals both quantitative and qualitative differences in freshly isolated and *in vitro* stimulated naïve CD8^+^ T cells between Kmit2d KO and WT mice. In freshly isolated naïve CD8^+^ T cells, Kmt2d KO mice had increased expression of mitochondrial proteins related to apoptosis and reduced expression of adhesion and cytoskeleton proteins. Post stimulation, the expression of some apoptosis-related genes (e.g., *Chchd10*) remained high and other apoptotic related genes (e.g., *Myc, Egr1, and NKbid*) became prominent in naïve CD8^+^ T cells of Kmt2d KO mice. Similarly, genes with reduced expression (e.g., *Rgcc, Actn1, Gmfg*, and *Rac2*) in freshly isolated naïve CD8^+^ T cells of Kmt2d KO mice remained lowly expressed post stimulation. In addition to these differences in gene expression levels, substantially fewer naïve CD8^+^ T cells of Kmt2d KO mice (66% in cluster one and 34% in cluster 2) were able to be activated post one-day stimulation based on the overall transcriptome changes compared to the naïve CD8^+^ T cells of Kmt2d WT mice (36% in cluster one and 64% in cluster 2), suggesting the ability to respond to activation signals, a pivotal function of naïve CD8^+^ T cells, is substantially impaired in the absence of Kmt2d.

Reduction of naïve CD8^+^ T cells in the peripheral lymphoid tissues and blood in Kmt2d deficient mice is partially due to reduced T cells in thymus. Flow cytometry analysis shows an increase of immature thymocytes in the absence of Kmt2d. However, the precise role of Kmt2d in T cell development requires further study. In contrast to the significant reduction of naïve CD8^+^ T cells, memory CD8^+^ T cells exhibit a much milder reduction. As mRNA levels of *Kmt2d* is much lower in memory than in naïve CD8^+^ T cells, it is possible that Kmt2d plays a limited role in memory CD8^+^ T cells. Nevertheless, it remains to be determined whether activation-induced increased apoptosis observed in naïve CD8^+^ T cells happens in memory CD8^+^ T cells in the absence of *Kmt2d* and if these surviving memory CD8^+^ T cells are less dependent on Kmt2d.

In conclusion, our findings reveal a critical role of Kmt2d in epigenetic regulation of gene expression involved in cell death by controlling the level of H3K4me1 at enhancers during naïve CD8^+^ T cell activation. Understanding how Kmt2d is recruited and catalyzes H3K4me1 at specific enhancers as well as its interaction with other regulators provides new insight into epigenetic regulation of gene expression during CD8^+^ T cell activation and differentiation.

## Methods

### Animals and cells

Kmt2d-floxed (Kmt2d^fl/fl^) mice were generated as described (9). CD4-Cre mice were obtained from Taconic, and OT-I, B6.SJL-PtpraJ mice from Jackson Laboratory. Kmt2d^fl/fl^ mice were crossed with CD4-Cre mice to generate the Kmt2d^fl/fl^-CD4-Cre^+^ (Kmt2d KO) strain. Kmt2d KO mice were further crossed with OT-I to generate Kmt2d KO-OT-I strain. All mice were maintained under specific pathogen-free conditions at the animal facility of National Institute on Aging and animal care was conducted in accordance with the guidelines of NIH.

### *In vitro* culture of naïve CD8^+^ T cells

Naïve CD8^+^ T cells were purified from spleen of Kmt2d WT or Kmt2d KO using StemCell CD8^+^ naïve T cell negative isolation kit (StemCell Technology) and purity of isolated cells were over 95%. Cells were cultured in RPMI-1640 with 10% FBS, 10mM HEPES, 0.11 nM beta-mercaptoethanol and 1x Pen/Strep/Glu from Thermo-Fisher with stimulations using plate coated anti-CD3 (2C11, 3µg/ml) and soluble anti-CD28 (37.51, 1μg/ml) (αCD3/CD28 antibodies) (Biolegend).

### Listeria infection

Listeria monocytogenes (10403S) with engineered OVA was a gift from Dr. Hao Shen of University of Pennsylvania and cultured in Brain Heart Infusion (BHI) media with 50 µg/mL erythromycin. Mice were immunized *via* tail vein injections of 5×10^4^ cfu of Listeria monocytogenes (38). All infection experiments were conducted under BSL-2 conditions with an approved protocol.

### Adoptive transfer

Naïve CD8^+^ T cells were isolated from the spleens of Kmt2d WT, Kmt2d KO, Kmt2d WT-OT1 or Kmt2d KO-OT1 mice by a kit from StemCell described above and adoptively transferred to CD45.1^+^ congenic (B6.SJL-PtpraJ) mice. Cells were pre-labeled with eFluor-450 cell proliferation tracker (eBioscience) for proliferation analysis. The mice were immunized with *Listeria monocytogenes* the next day and sacrificed or bled at indicated time points for FACS analysis.

### Flow cytometry analysis

The antibodies for flow cytometry used to detect surface and intracellular molecules (CD4, CD8, CD45.1, CD45.2, CD127, KLRG1, CD69, CD25, CD44, CD62L, CD95, CD24, TNF-α, and annexin V conjugated to various fluorescent dyes) were purchased from Biolegend/Tonbo and SIINFEKL-dextramer-PE from Immudex. For viability test, Ghost viability dye 510 (viability dye, Tonbo) was used (39). For activation-induced cytokine expression, naïve CD8^+^ T cells were stimulated by αCD3/CD28 antibodies for 72 hours and PMA (20 ng/ml), Ionomycin (1 μg/ml) (Sigma) and Golgi plug (BD) (1 μl/ml) were added 4 hours before collection. Stimulated cells were collected and permeabilized with Fix/Perm buffer (BD bioscience) and blocked with mouse/rat serum mixture (Stemcell Technology) to reduce nonspecific antibody staining. Permeabilized cells were stained in Perm buffer (contains saponin) with cytokine-specific or representative isotype control antibodies. For the apoptosis analysis, annexin V staining, and caspase 3/7 activity assay were performed. For annexin V staining, cells were stained with fluorophore conjugated annexin V and Ghost-viability dye along with cell surface antibodies. Caspase 3/7 activity was analyzed with CellEvent™ caspase 3/7 detection kit (ThermoFisher), which detects DEVDase activity of caspase-3/7 according to manufacturer’s protocol. Briefly, cells were incubated with CellEvent™ Caspase-3/7 Green Detection Reagent for 30 minutes in 37C incubator and then stained with other fluorescent conjugated cell surface antibodies including Ghost viability dye. The stained cells were acquired immediately on a flow cytometer. Flow cytometry data was acquired on a BD FACS CantoII or Accuri C6 flow cytometer and results were analyzed with FlowJo (10.4) (Tree Star).

### FACS cell sorting

Sorting was performed in MoFlo (Beckman Courter) and iCyt (Sony) cell sorters. Bead-based pre-enriched CD8^+^ T cells were stained with anti-CD8, CD44, and CD62L antibodies with variable fluorochromes to define naïve (CD62L^+^/CD44^-^), central memory (CD62L^+^/CD44^+^), and effector memory (CD62L^-^/CD44^+^) cells in the CD8^+^ population. SIINFEKL-dextramer (Immudex) was added to staining in the case of antigen-specific cell sorting.

### Proliferation assay

Proliferation assays were carried out with efluor450 proliferation tracker (eBioscience) incorporation assay. For efluor450 proliferation tracker assay, FACS-sorted or bead-enriched naïve CD8^+^ T cells were labeled with 1µM of efluor450 proliferation tracker according to manufacturer’s instructions. The labeled cells were cultured or adoptively transferred. Flow cytometric analysis was performed at 405 excitation and 450/50 band pass collection for each time point. Stained cells were analyzed using CantoII and FlowJo.

### Chromatin immunoprecipitation sequencing

Naïve CD8^+^ T cells from Kmt2d WT and Kmt2d KO mice were sorted by FACS and stimulated with α-CD3/CD28 *in vitro* for 24 hours. Human CD4^+^ naïve cells were sorted for a spike-in control. Cells were digested in digestion buffer (50mM Tris-HCl, 1mM CaCl2, 0.2% Triton X-100, 5 mM sodium butylate with proteinase inhibitor cocktail and 0.5 mM PMSF). Digested human CD4N cells were spiked into mouse cell digestions at the ratio of 10%. 0.2U MNase (ThermoFisher) was added per million cells and incubated at 37°C for 40 minutes and sonicated (Diagenode) with a power setting of 5 for 4 repeats of 30 seconds followed by 30 seconds break between each repeat in ice-cold water to obtain an average size between 100-300 bps. The sonicated products were dialyzed with 10000 MWCO Cassette G2 (ThermoFisher) in RIPA buffer for 2 hours at 4°C, and then incubated with Dynabeads Protein G (ThermoFisher) coated with 4 μg of anti-H3K4me1 antibody or non-specific IgG (Millipore) overnight. The precipitated beads were washed with RIPA, LiCl and TE buffer sequentially, and recovered overnight at 65°C with TE supplemented with 10% SDS and proteinase K (MBiotech). The ChIP DNA was purified with phenol/chloroform (Invitrogen) and precipitated.

For sequencing, adapters were ligated to the precipitated DNA fragments or the input DNA to construct a sequencing library according to the manufacturer’s protocol (Illumina). In short, the fragments were end repaired using DNA polymerases and kinases. The fragments were A tailed on the 3’ end and then T tailed illumine sequencing adapters, including unique indexes for each sample, were ligated to both ends of the DNA fragments. These library samples were then size selected (~250-400 bases) on a 4.5% agarose gel. An 18-cycle PCR amplification was performed to enrich for fragments containing adaptors on both ends. These amplified libraries were then tested for concentration and equal molar amounts of each were pooled and used to generate clusters onboard the Illumina HiSeq 2500 sequencer. These clusters were then sequenced, with Illumina HiSeq Rapid Run Reagents, for 68 cycles then for an additional 7 cycles to determine the index’s present in each unique cluster. The images generated were used to generate sequence reads: Using both lanes of the Illumina Rapid run flowcell we obtained ~500 million reads with 445 million reads passing the Illumina quality filter to be used for generating the actual sequencing reads. The index reads were between 10.2% and 14.8% for the 8 samples with an expected average of 12.5% for each. Of the passed filter reads, >94.5% had a quality score of Q30. Chip-Seq reads were filtered, adjusted for spiked-in human samples, and peak-called *via* SICER (40).

For ChIP-Seq of Kmt2d, sonicated chromatin was incubated with Dynabeads Protein A coated with 8 μg of anti-Kmt2d/Mll4 antibody (9) at 4°C overnight. Beads were washed with RIPA, RIPA with 300mM NaCl, LiCl buffer and PBS sequentially, and recovered overnight at 65°C with TE supplemented with 10% SDS and proteinase K. ChIPed DNA was purified using QIAquick PCR purification kit (Qiagen). Sequencing libraries were constructed using NEBNext^®^ Ultra™ II DNA Library Prep kit following manufacturer’s instruction. AMPure XP magnetic beads (Beckman Coulter) were used for size selection. PCR amplification of libraries was done for 15 cycles. Final libraries were sequenced on Illumina HiSeq 3000. For peak calling of Kmt2d, SICER (40) was used with the window size of 50 bp and the gap size of 50 bp. CPM values as reported were calculated *via* in-house Python scripts. Heatmap generation and analysis were done *via* Galaxy web platform (41).

### RNA-seq

Total RNA was extracted using RNaeasy Kit on QIAcube (Qiagen) from resting and stimulated sorted naïve CD8^+^ T cells (24 hours with anti-CD3/28 antibodies) from Kmt2d WT or Kmt2d *KO* mice. RNA samples were processed following the protocol for the Illumina TruSeq Stranded mRNA Sample Preparation Kit. Poly (A) tailed RNA was purified from 1 μg of total RNA, fragmented, and reverse-transcribed into cDNAs. Double strand cDNAs were adenylated at the 3’ ends and individually indexed, followed by limited-cycle (15) amplification. Paired-end sequencing (100 × 2 cycles) of multiplexed mRNA samples per lane was carried out on an Illumina HiSeq 2500 sequencer. Gene set enrichment analysis (GSEA) was performed using the R packages clusterProfiler and ReactomePA on genes ordered by indicated measures in defined analyses (42). GSEA annotation sets with normalized enrichment scores (NES) above 1.5 and q-value (FDR) below 10% were considered significant.

### scRNAseq library construction and data analysis

Naïve CD8^+^ T cells were isolated from Kmt2d WT or Kmt2d KO mice by cell sorting and half of them were stimulated with anti-CD3/28 antibodies for 24 hours. Both freshly sorted and stimulated naïve CD8^+^ T cells were counted for scRNAseq library construction using Single Cell 5’ VDJ library and Gel bead kits (10X Genomics) according to the manufacturer’s protocol. Briefly, resting and stimulated naïve CD8^+^ T cells were mixed with gel bead-in-emulsion (GEM) generation. After template-switching reverse transcription within GEMs, cDNA was amplified in bulk. Subsequent fragmentation, end repair, A-tailing, adaptor ligation, and sample index PCR generated P5- and P7- capped, cell- and sample-indexed single-cell 3’ gene expression libraries. PCR products were purified using 1.6X AmpureXP. Libraries were quantified using a bioanalyzer machine (Agilent) before sequencing. Sequencing was performed on the Illumina HiSeq 3000/4000 flow cell. Read 1, Read 2, and the sample index were sequenced to 28, 91, and 8 base pairs, respectively. Cellranger (6.0) was used for data processing.

The 2000 most variable genes in the dataset were chosen using the FindVariableFeatures function in Seurat using the “vst” method. Clustering was then done using the FindNeighbors and FindClusters functions in Seurat, using the 18 most variable principal components and 0.5 resolution using the Louvain Algorithm. Two clusters of resting (cluster 1) and stimulated (cluster 2) of naïve CD8 T^+^ cells were showed on UMAP projections. Differential Expression analysis was performed in each cluster using Seurat. Genes expressed in at least 30% of cells in each subset with at least at an average of 1.2-fold change were considered differentially expressed at a threshold of 5% False Discovery Rate (FDR) generated from the p-value using the Student’s T-test. Gene set enrichment analysis (GSEA) was performed using the R packages clusterProfiler and ReactomePA on genes ordered by indicated measures in defined analyses (42). GSEA was described above.

### Quantitative reverse transcription polymerase chain reaction for Kmt2d

Total RNA was extracted with RNaeasy Kit on QIAcube (Qiagen) from freshly isolated or stimulated naïve CD8^+^ T cells. RNA was reverse transcribed to cDNA using Super Script III (Invitrogen). Quantitative PCR was performed with SYBR green (Qiagen) by standard protocol. Gene primers are included: *Kmt2d* (F: 5’- GCTATCACCCGTACTGTGTCAACA-3’ and R: 5’-CACACACGATACACTCCACACAA-3’); *Angptl4* (F: 5’-GGGGACCTTAACTGTGCCAA-3’ and R: 5’-GCCGTGGGATAGAGTGGAAG-3’); *Bcl3* (F: 5’-CGGAGGCCCTTTACTACCAG-3’ and R: 5’-GGAGTAGGGGTGAGTAGGCA-3’); *Lmna* (F: 5’- GCAAAGTGCGTGAGGAGTTC-3’ and R: 5’-TCCTTGGAGTTGAGAAGAGCC-3’); *Rps6ka1*(F: 5’-TCAGGGGAAGAAGCTGGACT-3’ and R: 5’- TCAAACTGGGATGGATCGGC-3’)*; Axl* (F: 5’-GGTCGCTGTGAAGACCATGA-3’ and R: 5’- TGACGTTGGGGTGGTCAAAT-3’); *Hip1r* (F: 5’-GCGGGAACTGATGTCAACAA-3’ and R: 5’-AAGACATCTGGGACACTGCG-3’); *Lgals3* (F: 5’-ACTAATCAGGTGAGCGGCAC-3’ and R: 5’-CCTTGAGGGTTTGGGTTTCC-3’)*;* and *Gapdh* (F:5’-ATGTGTCCGTCGTGGATCTGA-3’ and R: 5’-CCTGCTTCACCACCTTCTTGA-3’) for normalization.

*Statistical analysis.* Student’s t-test was used to compare WT and KO mice at specified time points. Two-way Anova was used to compare the overall difference of a time course between WT and KO mice using Prism. *, ** and *** represent p<0.05, 0.01 and 0.001, respectively.

## Data availability statement

The RNAseq and ChIPseq data were deposited to NCBI SRA (PRJNA541991) and the scRNAseq data were deposited to GEO (GSE217656).

## Ethics statement

The animal study was reviewed and approved by NIA ACUC committee.

## Author contributions

JK and NPW conceived project. JK carried out most experiments, QY helped scRNAseq experiment, J-EL helped Kmt2d ChIPseq experiments, TN, XL, YK, and SD helped ATACseq and ChIPseq data analysis, RA and YZ helped RNAseq data analysis, JC helped scRNAseq data analysis, WP helped data analysis, KG provided Kmt2d KO mice and advice. JK and NPW wrote the manuscript. All authors contributed to the article and approved the submitted version.
