# Supplementary material for: Lysine methyltransferase Kmt2d regulates naive CD8+ T cell activation-induced survival
Source: Front Immunol. 2023 Jan 19;13:1095140. doi: 10.3389/fimmu.2022.1095140 (PMC9892454; doi:10.3389/fimmu.2022.1095140)
Supplement: Supplementary file 1 [file DataSheet_1.pdf]

Lysine methyltransferase Kmt2d regulates naive $CD8^+$ T cell activation-induced survival

Jaekwan Kim[1], Thomas Nguyen[1], Jeffrey Cifello[1], Raheel Ahmad[1], Yongqing Zhang[2], Qian Yang[1], Ji-Eun Lee[3], Xiang Li[4], Yan Kai[4], Supriyo De[2], Weiqun Peng[4], Kai Ge[3] and Nan-ping Weng[1*]

[1] Laboratory of Molecular Biology and Immunology, National Institute on Aging, National Institutes of Health,
[2] Laboratory of Genetics and Genomics, National Institute on Aging, National Institutes of Health,
[3] Laboratory of Endocrinology and Receptor Biology, National Institute of Diabetes, Digestive and Kidney Diseases, National Institutes of Health.
[4] Department of Physics, George Washington University

Supplementary Table 1. List of genes with decreased levels of H3K4me1 at enhancer and mRNA with and without direct Kmt2d binding

Supplementary Table 2. Antibodies used by flow cytometry

Supplementary Table 3. List of primers used for RT-PCR

Supplementary Figure 1. *Kmt2d* expression in naïve $CD8^+$ T cells and splenocyte counts in *Kmt2d* WT and KO mice.

Supplementary Figure 2 Impaired thymic development with Kmt2d depletion.

Supplementary Figure 3. Decreased expression of apoptotic genes upon stimulation.

Supplementary Table 1. List of genes with decreased levels of H3K4me1 at enhancer and mRNA with and without direct Kmt2d binding

Gene	Enhancer location			H3K4me1			RNA-seq (FPKM)			Kmt2d_ChIP WT	Heatmap Order
	Chromosome	Start	End	WT	KO	KO/WT (Log2)	WT	KO	KO/WT (Log2)		
Lgals1	chr15	78,926,591	78,926,841	5,719	2,468	-1.2	66.4	1.4	-5.6	1,693	1
Fut7	chr2	25,423,088	25,423,338	6,175	3,278	-0.9	2.7	1.0	-1.4	3,010	2
Limk1	chr5	134,678,153	134,678,403	4,144	2,226	-0.9	20.8	8.2	-1.3	15,236	3
Tbxa2r	chr10	81,328,543	81,328,793	3,274	1,983	-0.7	48.8	3.5	-3.8	28,215	4
Tnfsf14	chr17	57,194,070	57,194,320	4,559	2,711	-0.7	23.9	10.5	-1.2	22,384	5
Gpr146	chr5	139,389,729	139,389,979	5,222	2,347	-1.2	16.5	10.3	-0.7	14,860	6
Myo18a	chr11	77,801,110	77,801,360	4,517	2,590	-0.8	4.2	1.0	-2.1	3,574	7
Mdk	chr2	91,931,572	91,931,822	2,487	1,012	-1.3	3.7	2.3	-0.7	3,010	8
Padi2	chr4	140,906,168	140,906,418	1,450	607	-1.3	14.9	4.3	-1.8	8,276	9
Tspan4	chr7	141,476,266	141,476,516	4,849	2,752	-0.8	9.8	2.8	-1.8	1,317	10
Lta	chr17	35,205,117	35,205,367	3,440	1,416	-1.3	48.4	17.0	-1.5	4,703	11
Cxcr5	chr9	44,526,321	44,526,571	4,020	2,307	-0.8	2.7	1.0	-1.4	1,317	12
Tnf	chr17	35,201,951	35,202,201	3,191	1,861	-0.8	56.7	34.3	-0.7	4,514	13
Rasal1	chr5	120,649,105	120,649,355	1,036	283	-1.9	5.7	2.5	-1.2	5,079	14
Angptl4	chr17	33,781,476	33,781,726	1,865	809	-1.2	5.8	2.7	-1.1	4,703	15
Lmna	chr3	88,493,240	88,493,490	3,191	1,012	-1.7	2.7	1.5	-0.9	1,693	16
Cyp2s1	chr7	25,816,432	25,816,682	2,238	1,093	-1.0	4.4	1.0	-2.1	29,908	17
Rgs14	chr13	55,369,623	55,369,873	1,492	688	-1.1	32.8	11.2	-1.6	16,929	18
Gngt2	chr11	95,842,101	95,842,351	3,232	1,376	-1.2	9.0	5.5	-0.7	96,683	19
Bcl3	chr7	19,822,733	19,822,983	1,036	526	-1.0	19.4	9.7	-1.0	8,653	20
Nkg7	chr7	43,436,926	43,437,176	4,020	2,388	-0.8	222.2	47.8	-2.2	5,079	21
Hvcn1	chr5	122,210,120	122,210,370	4,186	1,335	-1.6	15.7	7.0	-1.2	49,658	22
B3galt4	chr17	33,951,402	33,951,652	1,036	324	-1.7	19.5	9.9	-1.0	7,524	23
Sept9	chr11	117,266,065	117,266,315	1,823	486	-1.9	108.8	54.2	-1.0	13,167	24
Tha1	chr11	117,873,356	117,873,606	2,321	1,214	-0.9	8.8	4.7	-0.9	3,386	25
Susd3	chr13	49,248,094	49,248,344	2,031	1,174	-0.8	11.9	4.6	-1.4	3,574	26
Flt3l	chr7	45,136,345	45,136,595	1,865	1,012	-0.9	21.6	11.7	-0.9	6,207	27
Thy1	chr9	44,043,288	44,043,538	2,777	850	-1.7	422.3	54.7	-2.9	5,079	28
Vipr1	chr9	121,642,545	121,642,795	787	202	-2.0	29.1	17.6	-0.7	10,534	29
Rab11fip4	chr11	79,643,200	79,648,400	5,387	2,509	-1.1	8.7	2.6	-1.7	16,929	30
Abhd8	chr8	71,463,574	71,463,824	2,611	1,214	-1.1	55.5	34.2	-0.7	7,712	31
Wdfy1	chr1	79,761,666	79,761,916	995	364	-1.4	10.4	5.6	-0.9	7,336	32
Pstpip1	chr9	56,089,821	56,090,071	2,114	1,214	-0.8	61.1	33.6	-0.9	7,148	33
Slc17a9	chr2	180,725,183	180,725,433	3,232	1,740	-0.9	42.1	11.0	-1.9	4,891	34
Vamp5	chr6	72,380,401	72,380,651	829	202	-2.0	5.3	1.8	-1.5	7,712	35
Ehd3	chr17	73,804,685	73,804,935	2,196	1,133	-1.0	48.9	28.1	-0.8	5,643	36
Dbp	chr7	45,705,215	45,705,465	1,285	567	-1.2	6.6	4.1	-0.7	4,514	37
Sema4b	chr7	80,186,757	80,187,007	2,196	647	-1.8	12.3	6.6	-0.9	19,751	38
Trp53i13	chr11	77,513,223	77,513,473	414	1	-8.7	13.5	8.3	-0.7	14,860	39
Bspry	chr4	62,479,850	62,480,100	787	445	-0.8	3.9	1.4	-1.5	15,236	40
Il18r1	chr1	40,465,760	40,466,010	3,232	1,740	-0.9	18.1	3.0	-2.6	3,010	41
Acot7	chr4	152,178,033	152,178,283	580	1	-9.2	58.7	36.4	-0.7	10,534	42
Hcst	chr7	30,419,737	30,419,987	1,658	769	-1.1	125.6	56.2	-1.2	4,891	43
Anxa6	chr11	55,033,332	55,033,582	1,326	283	-2.2	144.7	67.3	-1.1	28,967	44
Sh2d3c	chr2	32,720,918	32,721,168	1,658	1,012	-0.7	52.3	28.1	-0.9	3,386	45
Nudt14	chr12	112,942,084	112,942,334	1,575	728	-1.1	36.4	17.5	-1.1	6,395	46
Dag1	chr9	108,263,767	108,264,017	663	1	-9.4	13.8	8.3	-0.7	13,919	47
Rps6ka1	chr4	133,887,700	133,887,950	207	1	-7.7	67.6	37.1	-0.9	31,225	48
Ap3m2	chr8	22,805,618	22,805,868	1,699	526	-1.7	16.0	9.9	-0.7	22,572	49
Acsf2	chr11	94,601,752	94,602,002	332	121	-1.4	17.0	9.8	-0.8	4,703	50

Cdc25b	chr2	131,186,796	131,187,046	622	202	-1.6	55.7	22.5	-1.3	7,336	51
Tec	chr5	72,868,340	72,868,590	414	1	-8.7	12.9	5.5	-1.2	7,148	52
Tex9	chr9	72,491,871	72,492,121	746	364	-1.0	1.8	1.0	-0.9	3,762	53
Agfg2	chr5	137,684,684	137,684,934	912	486	-0.9	10.2	6.2	-0.7	17,305	54
Rab3d	chr9	21,918,051	21,918,301	1,036	445	-1.2	3.6	1.8	-1.0	15,424	55
Fhl3	chr4	124,700,564	124,700,814	456	162	-1.5	19.1	6.4	-1.6	13,167	56
Cpm	chr10	117,629,350	117,629,600	414	202	-1.0	7.9	2.2	-1.8	15,048	57
Rmnd5b	chr11	51,635,649	51,635,899	1,409	324	-2.1	32.4	19.1	-0.8	9,405	58
Appl2	chr10	83,648,615	83,648,865	1,036	121	-3.1	20.6	7.8	-1.4	9,029	59
Icosl	chr10	78,069,160	78,069,410	2,155	769	-1.5	2.1	1.0	-1.1	11,474	60
Nbeal2	chr9	110,654,086	110,654,336	1,409	486	-1.5	35.0	9.8	-1.8	10,346	61
Smpd5	chr15	76,294,246	76,294,496	1,077	607	-0.8	10.3	4.0	-1.4	2,633	62
Adgre5	chr8	83,741,118	83,741,368	207	1	-7.7	148.6	50.7	-1.6	6,772	63
Fam214b	chr4	43,046,082	43,046,332	1,989	850	-1.2	4.4	2.5	-0.8	12,603	64
Lzts2	chr19	45,018,060	45,018,310	1,036	1	-10.0	4.5	1.6	-1.5	1,505	65
Sh3pxd2a	chr19	47,315,000	47,319,400	7,211	2,833	-1.3	3.7	1.0	-1.9	6,019	66
Il10ra	chr9	45,269,047	45,269,297	2,321	890	-1.4	25.6	3.9	-2.7	1,693	67
Abca2	chr2	25,428,480	25,428,730	1,036	283	-1.9	31.7	18.2	-0.8	9,217	68
Kif1b	chr4	149,307,687	149,307,937	953	283	-1.8	20.6	9.4	-1.1	15,800	69
Als2cl	chr9	110,880,049	110,880,299	456	81	-2.5	158.7	60.7	-1.4	18,434	70
Serp2	chr14	76,556,634	76,556,884	1,036	202	-2.4	10.2	3.6	-1.5	20,691	71
Tsc22d3	chrX	140,600,512	140,600,762	1,823	809	-1.2	69.1	40.5	-0.8	12,227	72
Ahnak	chr19	8,989,135	8,989,385	1,533	283	-2.4	13.5	3.2	-2.1	28,967	73
Hist1h4a	chr13	23,761,177	23,761,427	1,409	607	-1.2	3.2	1.6	-1.0	16,553	74
Serpinb6b	chr13	32,965,317	32,965,567	1,616	971	-0.7	27.1	13.7	-1.0	2,069	75
Gsn	chr2	35,256,231	35,256,481	1,368	850	-0.7	8.0	2.1	-1.9	13,355	76
Lax1	chr1	133,764,100	133,766,100	2,528	1,093	-1.2	29.9	10.6	-1.5	9,969	77
Baiap2	chr11	119,943,013	119,943,263	290	1	-8.2	12.8	5.3	-1.3	14,860	78
Rnf32	chr5	29,195,749	29,195,999	829	445	-0.9	5.3	2.3	-1.2	5,831	79
Ccdc102a	chr8	94,913,900	94,915,900	16,618	8,134	-1.0	9.2	1.0	-3.2	2,822	80
March3	chr18	56,925,509	56,925,759	746	202	-1.9	2.6	1.3	-1.0	30,096	81
Dennd4c	chr4	86,748,319	86,748,569	1,782	405	-2.1	13.0	6.9	-0.9	16,365	82
AI467606	chr7	127,091,207	127,091,457	912	445	-1.0	104.7	60.1	-0.8	33,858	83
Rilpl2	chr5	124,477,995	124,478,245	580	1	-9.2	96.5	51.9	-0.9	3,198	84
Sntb2	chr8	106,935,517	106,935,767	912	243	-1.9	5.0	2.7	-0.9	11,098	85
Il2rb	chr15	78,494,822	78,495,072	829	405	-1.0	139.7	81.7	-0.8	1,881	86
Slc25a53	chrX	137,038,226	137,038,476	456	81	-2.5	7.3	1.7	-2.2	4,326	87
Rgs10	chr7	128,418,055	128,418,305	207	1	-7.7	130.0	51.6	-1.3	3,762	88
Hpcal1	chr12	17,690,604	17,690,854	912	121	-2.9	72.9	42.9	-0.8	12,038	89
Lat	chr7	126,369,417	126,369,667	622	364	-0.8	442.4	235.8	-0.9	19,374	90
Pik3r5	chr11	68,431,883	68,432,133	207	1	-7.7	49.1	27.7	-0.8	10,910	91
Gmfg	chr7	28,440,789	28,441,039	1,160	647	-0.8	112.9	52.5	-1.1	15,989	92
Abcb9	chr5	124,095,651	124,095,901	2,238	769	-1.5	4.3	1.0	-2.1	3,574	93
Usp28	chr9	48,998,900	49,000,900	1,326	567	-1.2	52.0	21.6	-1.3	22,572	94
Mcoln2	chr3	146,149,710	146,149,960	953	121	-3.0	7.8	4.0	-1.0	3,198	95
Oasl2	chr5	115,181,710	115,183,710	1,575	607	-1.4	3.9	2.3	-0.7	3,198	96
Whsc1l1	chr8	25,601,452	25,601,702	870	202	-2.1	85.6	53.4	-0.7	11,850	97
S100a10	chr3	93,554,992	93,555,242	83	1	-6.4	522.2	231.5	-1.2	10,722	98
Socs3	chr11	117,969,166	117,969,416	456	1	-8.8	28.8	8.4	-1.8	9,969	99
Orai2	chr5	136,170,558	136,170,808	539	324	-0.7	85.5	29.2	-1.6	12,415	100
Gtf2i	chr5	134,314,758	134,315,008	953	445	-1.1	123.4	56.8	-1.1	7,336	101
Sh3bp1	chr15	78,899,641	78,899,891	290	1	-8.2	70.3	40.2	-0.8	8,653	102
Dlg4	chr11	70,025,200	70,031,900	49,813	24,564	-1.0	8.3	1.0	-3.1	29,344	103
Adam19	chr11	46,055,928	46,056,178	1,492	607	-1.3	9.8	5.1	-0.9	3,386	104

Ctsd	chr7	142,387,842	142,388,092	2,072	728	-1.5	220.2	137.4	-0.7	11,662	105
Map7d1	chr4	126,256,183	126,256,433	166	1	-7.4	41.4	23.2	-0.8	9,593	106
Tecpr1	chr5	144,223,492	144,223,742	622	1	-9.3	65.9	38.8	-0.8	18,810	107
Tpst2	chr5	112,276,471	112,276,721	332	1	-8.4	79.1	37.7	-1.1	23,136	108
Cerk	chr15	86,186,081	86,186,331	3,440	1,942	-0.8	15.9	8.8	-0.8	2,257	109
Cpt1a	chr19	3,323,184	3,323,434	332	162	-1.0	23.2	14.4	-0.7	11,474	110
Rnf122	chr8	31,111,631	31,111,881	746	405	-0.9	2.6	1.1	-1.2	4,891	111
Hid1	chr11	115,367,628	115,367,878	1,243	445	-1.5	22.7	10.7	-1.1	6,395	112
Cdc42ep3	chr17	79,354,931	79,355,181	83	1	-6.4	10.9	4.8	-1.2	11,474	113
Fam46a	chr9	85,326,937	85,327,187	124	1	-7.0	10.7	4.7	-1.2	6,772	114
Arhgap26	chr18	38,992,965	38,993,215	580	1	-9.2	5.5	2.6	-1.1	10,910	115
Wbscr27	chr5	134,932,178	134,932,428	373	1	-8.5	6.5	2.6	-1.3	6,395	116
Pet117	chr2	144,368,756	144,369,006	746	81	-3.2	6.2	1.8	-1.8	7,712	117
Ppp3cc	chr14	70,289,323	70,289,573	207	1	-7.7	38.1	19.2	-1.0	10,346	118
Rgcc	chr14	79,301,467	79,301,717	332	81	-2.0	75.8	45.5	-0.7	16,177	119
Lats2	chr14	57,746,039	57,746,289	332	1	-8.4	13.9	7.7	-0.9	6,584	120
Socs1	chr16	10,785,428	10,785,678	290	1	-8.2	110.4	50.4	-1.1	24,453	121
Crip1	chr12	111,681,000	111,684,400	1,119	1	-10.1	430.7	268.7	-0.7	17,681	122
Axl	chr7	25,788,682	25,788,932	1,658	890	-0.9	2.3	1.3	-0.9	22,384	123
Cd1d1	chr3	86,999,196	86,999,446	332	1	-8.4	13.4	8.3	-0.7	4,703	124
Axin2	chr11	108,920,236	108,920,486	1,285	405	-1.7	11.7	5.4	-1.1	21,255	125
Notch1	chr2	26,503,698	26,503,948	414	40	-3.4	36.4	21.9	-0.7	14,296	126
Slc43a2	chr11	75,531,933	75,532,183	456	1	-8.8	23.5	10.0	-1.2	11,474	127
Akna	chr4	63,372,000	63,374,600	6,465	2,509	-1.4	106.0	63.9	-0.7	9,969	128
Tob1	chr11	94,211,359	94,211,609	249	1	-8.0	15.2	9.3	-0.7	19,751	129
Plec	chr15	76,209,002	76,209,252	83	1	-6.4	33.8	18.7	-0.9	24,829	130
Arid3a	chr10	81,108,600	81,110,600	2,196	40	-5.8	2.3	1.4	-0.7	18,058	131
Smap2	chr4	121,017,132	121,017,382	580	324	-0.8	151.3	69.3	-1.1	14,484	132
Actn1	chr12	80,260,123	80,260,373	83	1	-6.4	154.3	60.5	-1.4	14,296	133
Frmd4a	chr2	4,400,899	4,401,149	580	1	-9.2	2.5	1.0	-1.3	5,267	134
Ostf1	chr19	18,631,753	18,632,003	539	121	-2.1	94.4	58.9	-0.7	30,284	135
Smpdl3b	chr4	133,029,100	133,031,100	4,351	2,266	-0.9	1.8	1.0	-0.9	12,415	136
Glipr2	chr4	43,957,577	43,957,827	373	40	-3.2	121.2	49.7	-1.3	6,584	137
Acap1	chr11	69,890,000	69,892,000	14,422	7,932	-0.9	90.5	42.0	-1.1	3,762	138
Galnt10	chr11	57,645,320	57,645,570	83	1	-6.4	13.8	8.4	-0.7	6,960	139
Gse1	chr8	120,407,600	120,410,400	3,067	1	-11.6	15.1	7.9	-0.9	8,276	140
Vps37b	chr5	123,991,800	123,994,200	2,901	1	-11.5	77.1	44.5	-0.8	21,443	141
Id3	chr4	136,109,500	136,111,500	6,258	2,630	-1.3	75.3	26.3	-1.5	11,474	142
Sgk1	chr10	21,994,467	21,994,717	124	1	-7.0	13.7	7.6	-0.8	8,653	143
Cxxc5	chr18	35,865,000	35,869,400	9,242	3,925	-1.2	12.5	7.5	-0.7	3,386	144
Nsg2	chr11	31,733,400	31,735,400	373	1	-8.5	49.6	25.0	-1.0	3,574	145
Il10rb	chr16	91,564,100	91,566,100	2,321	1	-11.2	45.5	24.2	-0.9	10,157	146
Cmtm7	chr9	114,804,000	114,806,000	414	81	-2.4	68.3	40.9	-0.7	9,217	147
Fam53b	chr7	132,741,800	132,743,800	2,279	1,052	-1.1	39.0	24.3	-0.7	21,067	148
Gramd4	chr15	86,134,000	86,136,000	1,865	1	-10.9	95.1	58.4	-0.7	14,672	149
Neurl3	chr1	36,087,200	36,091,400	9,117	2,104	-2.1	77.4	18.2	-2.1	40,818	150
Phf21b	chr15	84,830,900	84,832,900	1,077	1	-10.1	2.9	1.0	-1.5	11,474	151
Qprt	chr7	127,117,963	127,119,963	2,404	1,254	-0.9	10.9	6.2	-0.8	18,810	152
Cxcr4	chr1	128,399,700	128,401,700	373	81	-2.2	38.0	23.6	-0.7	13,919	153
Adcy7	chr8	88,282,779	88,283,029	829	1	-9.7	100.8	16.7	-2.6	6,584	154
Rcsd1	chr1	165,662,000	165,664,400	2,611	1	-11.4	62.9	32.6	-0.9	30,472	155
Sgk3	chr1	9,773,000	9,779,300	1,326	1	-10.4	3.0	1.7	-0.8	15,612	156
Pear1	chr3	87,761,200	87,766,443	33,361	18,736	-0.8	8.4	1.6	-2.4	10,534	157
Eng	chr2	32,667,400	32,669,400	953	1	-9.9	10.2	2.8	-1.9	19,562	158

Rgs12	chr5	34,998,889	34,999,139	912	1	-9.8	3.1	1.9	-0.7	12,415	159
Btbd11	chr10	85,463,300	85,467,300	2,487	1	-11.3	10.4	3.9	-1.4	13,167	160
Tns1	chr1	73,962,200	73,965,000	1,658	1	-10.7	3.5	1.8	-0.9	2,445	161
Sipa1l2	chr8	125,869,300	125,871,300	6,631	2,549	-1.4	3.1	1.7	-0.9	12,038	162
Snai3	chr8	122,450,200	122,453,600	2,404	1	-11.2	5.2	1.0	-2.4	5,267	163
Itfg3	chr17	26,546,600	26,550,100	1,989	1	-11.0	23.4	10.7	-1.1	17,681	164
Gpr68	chr12	100,420,100	100,422,100	1,741	1	-10.8	76.2	29.7	-1.4	941	165
Fam101b	chr11	76,319,000	76,321,000	1,368	1	-10.4	46.6	23.2	-1.0	7,336	166
Syt11	chr3	88,226,779	88,240,800	2,362	890	-1.4	11.1	5.9	-0.9	6,960	167
Il12rb1	chr8	70,808,262	70,808,512	2,238	1,174	-0.9	24.4	7.6	-1.7	0	1
Baiap3	chr17	25,256,152	25,256,402	1,865	850	-1.1	11.6	1.0	-3.5	0	2
Bcl9l	chr9	44,498,920	44,499,170	2,196	890	-1.3	42.7	14.4	-1.6	0	3
Cd79b	chr11	106,314,470	106,314,720	5,056	1,902	-1.4	14.7	1.0	-3.9	0	4
Cd72	chr4	43,454,507	43,454,757	5,180	2,954	-0.8	14.2	2.1	-2.8	0	5
Podnl1	chr8	84,125,742	84,125,992	4,641	1,578	-1.6	5.1	1.0	-2.4	0	6
Grifin	chr5	140,564,969	140,565,219	4,061	1,497	-1.4	2.5	1.3	-0.9	0	7
Tesc	chr5	118,027,674	118,027,924	663	1	-9.4	6.6	3.4	-1.0	0	8
Sytl1	chr4	133,263,024	133,263,274	4,393	1,781	-1.3	19.3	1.3	-3.9	0	9
Ncf4	chr15	78,244,663	78,244,913	5,470	2,995	-0.9	26.7	6.3	-2.1	0	10
Pltp	chr2	164,857,647	164,857,897	2,404	769	-1.6	4.4	1.0	-2.1	0	11
Ncf1	chr5	134,229,476	134,229,726	4,600	2,347	-1.0	41.8	1.0	-5.4	0	12
Afmid	chr11	117,825,772	117,826,022	4,061	1,861	-1.1	7.7	4.7	-0.7	0	13
Gpr55	chr1	85,960,991	85,961,241	5,760	3,359	-0.8	1.9	1.0	-0.9	0	14
Tubb3	chr8	123,411,369	123,411,619	2,114	728	-1.5	3.7	1.0	-1.9	0	15
Cd24a	chr10	43,579,047	43,579,297	1,285	486	-1.4	4.6	2.8	-0.7	0	16
Lag3	chr6	124,911,624	124,911,874	2,777	971	-1.5	28.6	15.7	-0.9	0	17
Slfn5	chr11	82,951,949	82,952,199	2,735	1,457	-0.9	7.9	3.4	-1.2	0	18
Ecm1	chr3	95,739,455	95,739,705	5,346	2,468	-1.1	6.2	3.5	-0.8	0	19
Nfam1	chr15	83,033,220	83,033,470	3,564	1,457	-1.3	4.0	1.0	-2.0	0	20
Tpm1	chr9	67,049,028	67,049,278	1,409	728	-1.0	39.2	24.4	-0.7	0	21
Actn2	chr13	12,340,632	12,340,882	1,658	607	-1.4	4.0	1.0	-2.0	0	22
Cbr3	chr16	93,683,051	93,683,301	2,362	1,052	-1.2	2.0	1.0	-1.0	0	23
S100a4	chr3	90,603,648	90,603,898	4,890	2,630	-0.9	6.7	1.0	-2.7	0	24
Icam4	chr9	21,314,216	21,316,216	16,245	8,984	-0.9	1.7	1.0	-0.8	0	25
Nme4	chr17	26,095,372	26,095,622	1,823	890	-1.0	19.9	7.1	-1.5	0	26
Rgs11	chr17	26,202,814	26,203,064	2,942	1,659	-0.8	5.7	3.2	-0.8	0	27
Gna15	chr10	81,524,058	81,524,308	4,890	1,254	-2.0	5.3	1.0	-2.4	0	28
Chn2	chr6	54,039,832	54,040,082	953	243	-2.0	1.8	1.0	-0.9	0	29
Rinl	chr7	28,788,834	28,789,084	2,445	1,133	-1.1	77.9	44.3	-0.8	0	30
Tbx21	chr11	97,115,203	97,115,453	995	405	-1.3	16.6	6.5	-1.4	0	31
Rcn3	chr7	45,092,093	45,092,343	2,321	890	-1.4	54.3	29.5	-0.9	0	32
Fry	chr5	150,259,842	150,260,092	3,067	1,740	-0.8	1.7	1.0	-0.8	0	33
Slpi	chr2	164,356,268	164,356,518	3,854	2,347	-0.7	3.3	1.0	-1.7	0	34
Tirap	chr9	35,199,841	35,200,091	995	324	-1.6	1.7	1.0	-0.8	0	35
Nacc2	chr2	26,122,621	26,122,871	580	324	-0.8	2.5	1.0	-1.3	0	36
Irf7	chr7	141,266,371	141,266,621	1,533	526	-1.5	32.7	18.5	-0.8	0	37
Bin1	chr18	32,377,144	32,377,394	2,445	728	-1.7	28.4	17.1	-0.7	0	38
Fbxo17	chr7	28,716,663	28,716,913	1,450	526	-1.5	3.2	1.5	-1.1	0	39
Galnt6	chr15	100,729,313	100,729,563	1,492	283	-2.4	35.6	15.8	-1.2	0	40
Ccl4	chr11	83,662,536	83,662,786	3,854	2,185	-0.8	545.0	209.9	-1.4	0	41
Pygm	chr19	6,384,216	6,384,466	1,906	1,174	-0.7	2.6	1.3	-1.0	0	42
Zcchc18	chrX	136,993,020	136,993,270	539	162	-1.7	3.9	1.5	-1.4	0	43
Marveld2	chr13	100,616,885	100,617,135	1,409	809	-0.8	2.8	1.0	-1.5	0	44

Thra	chr11	98,741,651	98,741,901	41	1	-5.4	30.1	13.9	-1.1	0	45
Slc35g2	chr9	100,570,907	100,571,157	2,196	769	-1.5	1.6	1.0	-0.7	0	46
Adap1	chr5	139,325,407	139,325,657	912	486	-0.9	6.0	3.2	-0.9	0	47
Kcnip2	chr19	45,812,209	45,812,459	3,523	1,942	-0.9	3.4	1.6	-1.1	0	48
Rnf135	chr11	80,183,727	80,183,977	995	526	-0.9	4.8	2.7	-0.8	0	49
S100a11	chr3	93,520,286	93,520,536	2,279	1,416	-0.7	315.8	150.9	-1.1	0	50
Elf4	chrX	48,463,073	48,463,323	2,072	647	-1.7	44.3	26.2	-0.8	0	51
Cuedc1	chr11	88,099,066	88,099,316	456	243	-0.9	2.9	1.5	-1.0	0	52
Lypd6b	chr2	49,787,567	49,787,817	1,119	526	-1.1	4.6	2.1	-1.2	0	53
App	chr16	85,173,636	85,173,886	1,450	243	-2.6	4.0	2.4	-0.7	0	54
Dapl1	chr2	59,484,515	59,484,765	2,611	1,497	-0.8	193.0	71.2	-1.4	0	55
Lysmd2	chr9	75,625,599	75,625,849	787	1	-9.6	13.6	7.4	-0.9	0	56
Zbtb32	chr7	30,592,868	30,593,118	1,906	1,133	-0.8	15.6	2.4	-2.7	0	57
Irgm2	chr11	58,214,809	58,215,059	539	121	-2.1	111.7	57.5	-1.0	0	58
Lgals3	chr14	47,373,759	47,374,009	3,605	1,133	-1.7	2.3	1.0	-1.2	0	59
Osbpl5	chr7	143,740,240	143,740,490	2,859	1,093	-1.4	17.1	7.0	-1.3	0	60
Cd9	chr6	125,494,639	125,494,889	1,077	162	-2.7	44.2	8.0	-2.5	0	61
F2rl1	chr13	95,525,146	95,525,396	2,818	1,133	-1.3	33.3	7.4	-2.2	0	62
St3gal6	chr16	58,523,159	58,523,409	332	202	-0.7	7.8	3.2	-1.3	0	63
Reck	chr4	43,875,381	43,875,631	622	162	-1.9	3.4	1.0	-1.8	0	64
Gstt1	chr10	75,798,473	75,798,723	3,067	1,821	-0.8	2.7	1.0	-1.5	0	65
Pak6	chr2	118,677,113	118,677,363	2,114	607	-1.8	2.1	1.0	-1.1	0	66
Hddc3	chr7	80,342,987	80,343,237	1,616	971	-0.7	6.2	2.8	-1.2	0	67
Tyrobp	chr7	30,413,634	30,413,884	1,450	364	-2.0	3.3	1.0	-1.7	0	68
Unc93b1	chr19	3,935,052	3,935,302	2,321	809	-1.5	11.0	3.4	-1.7	0	69
Neu3	chr7	99,828,291	99,828,541	2,279	1,335	-0.8	5.6	2.7	-1.1	0	70
Ccl5	chr11	83,530,401	83,530,651	2,611	1,457	-0.8	73.3	16.8	-2.1	0	71
Sema4a	chr3	88,458,787	88,459,037	580	1	-9.2	16.8	3.6	-2.2	0	72
Golm1	chr13	59,675,706	59,675,956	456	1	-8.8	30.0	11.6	-1.4	0	73
Ap1m2	chr9	21,312,215	21,312,465	663	1	-9.4	1.7	1.0	-0.8	0	74
Hopx	chr5	77,096,988	77,100,900	829	1	-9.7	27.9	10.8	-1.4	0	75
Pde2a	chr7	101,450,434	101,450,684	912	243	-1.9	122.1	44.6	-1.5	0	76
Calhm2	chr19	47,437,800	47,439,800	3,398	1,497	-1.2	4.0	2.2	-0.9	0	77
Lcn2	chr2	32,387,645	32,387,895	414	162	-1.4	3.2	1.0	-1.7	0	78
Hip1r	chr5	124,452,400	124,454,400	2,362	364	-2.7	42.0	5.9	-2.8	0	79
Uba7	chr9	107,963,400	107,965,400	1,077	1	-10.1	56.5	33.7	-0.7	0	80
Lrrc75b	chr10	75,555,600	75,557,830	2,362	931	-1.3	6.5	1.6	-2.1	0	81
Crabp2	chr3	87,944,282	87,946,282	4,186	2,428	-0.8	10.7	1.0	-3.4	0	82
Emilin1	chr5	30,974,200	30,976,200	290	1	-8.2	4.4	1.0	-2.1	0	83
Haao	chr17	84,389,331	84,390,883	663	283	-1.2	6.4	3.8	-0.7	0	84
Ramp1	chr1	91,208,400	91,213,800	3,232	1,133	-1.5	5.1	1.6	-1.7	0	85
Vav2	chr2	27,296,000	27,298,000	5,719	2,347	-1.3	5.9	3.1	-0.9	0	86
Rhoq	chr17	87,193,000	87,195,000	1,160	1	-10.2	2.7	1.5	-0.9	0	87
Dtx4	chr19	12,485,800	12,496,600	6,092	3,763	-0.7	2.1	1.0	-1.1	0	88
Xaf1	chr11	74,492,900	74,494,900	1,285	1	-10.3	28.3	15.3	-0.9	0	89
Plekha6	chr1	133,306,100	133,308,100	1,160	1	-10.2	2.6	1.1	-1.3	0	90

Supplementary Table 2. Antibodies used by flow cytometry

Antigen	Fluorochrome	Company	Clone
CD3	FITC	Tonbo	17A2
CD4	BV421	Biolegend	GK1.5
CD8	APC-cy7	Tonbo	53-6.7
CD45.1	PE	Biolegend	A20
CD45.2	FITC	Biolegend	104
CD127	FITC	Biolegend	A7R34
KLRG1	PE-Cy7	Biolegend	2F1/KLRG1
CD69	APC	Biolegend	H1.2F3
CD25	PerCP-Cy5.5	Tonbo	PC61.5
CD44	PerCP-Cy5.5	Tonbo	IM7
CD62L	APC	Tonbo	MEL-14
CD95	APC	Biolegend	SA367H8
CD24	PE-Cy7	Biolegend	30-F1
TCR-b	PerCP-Cy5.5	Biolegend	H57-597
TNF-a	PE-Cy7	Biolegend	MP6-XT22
Annexin V	FITC	Biolegend	-
SIINFEKL-MHCI-dextramer	PE	Immudex	-

Supplementary Table 3 List of primers used for RT-PCR		
Gene	Forward	Reverse
Angptl4	GGGGACCTTAACTGTGCCAA	GCCGTGGGATAGAGTGGAAG
Axl	GGTCGCTGTGAAGACCATGA	TGACGTTGGGGTGGTCAAAT
Hip1r	GCGGGAACTGATGTCAACAA	AAGACATCTGGGACACTGCG
Kmt2d	GCTATCACCCGTACTGTGTCAACA	CACACACGATACACTCCACACAA
Lgals3	ACTAATCAGGTGAGCGGCAC	CCTTGAGGGTTTGGGTTTCC
Lmna	GCAAAGTGCGTGAGGAGTTC	TCCTTGGAGTTGAGAAGAGCC
Rps6ka1	TCAGGGGAAGAAGCTGGACT	TCAAACTGGGATGGATCGGC
Gapdh	GCTATCACCCGTACTGTGTCAACA	CACACACGATACACTCCACACAA

a

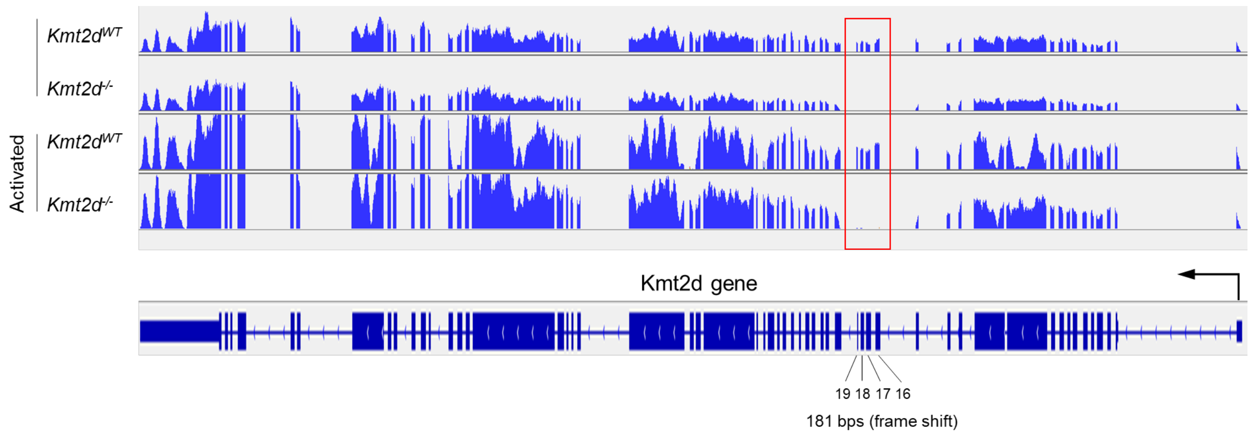

b

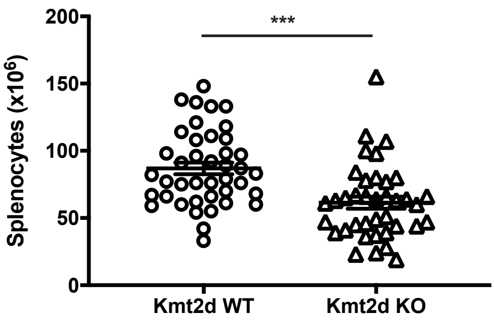

Supplementary Figure 1. *Kmt2d* expression in naïve CD8$^+$ T cells and splenocyte counts in *Kmt2d* WT and KO mice. a. *Kmt2d* expression in naïve CD8$^+$ T cells. mRNA expression of *Kmt2d* gene in naïve CD8$^+$ T cells from spleen of Kmt2d WT or Kmt2d KO mice. FACS-sorted cells were lysed freshly or after anti-CD3/anti-CD28 antibody stimulation (24 hours) and then performed RNA-seq. Red rectangle indicates deletion site (exon16-19) in Kmt2d KO mice. **b.** Live cell count from spleen. Freshly isolated splenocytes were counted by hemocytometer with trypan blue staining (N=41 for Kmt2d WT; N=38 for Kmt2d KO).

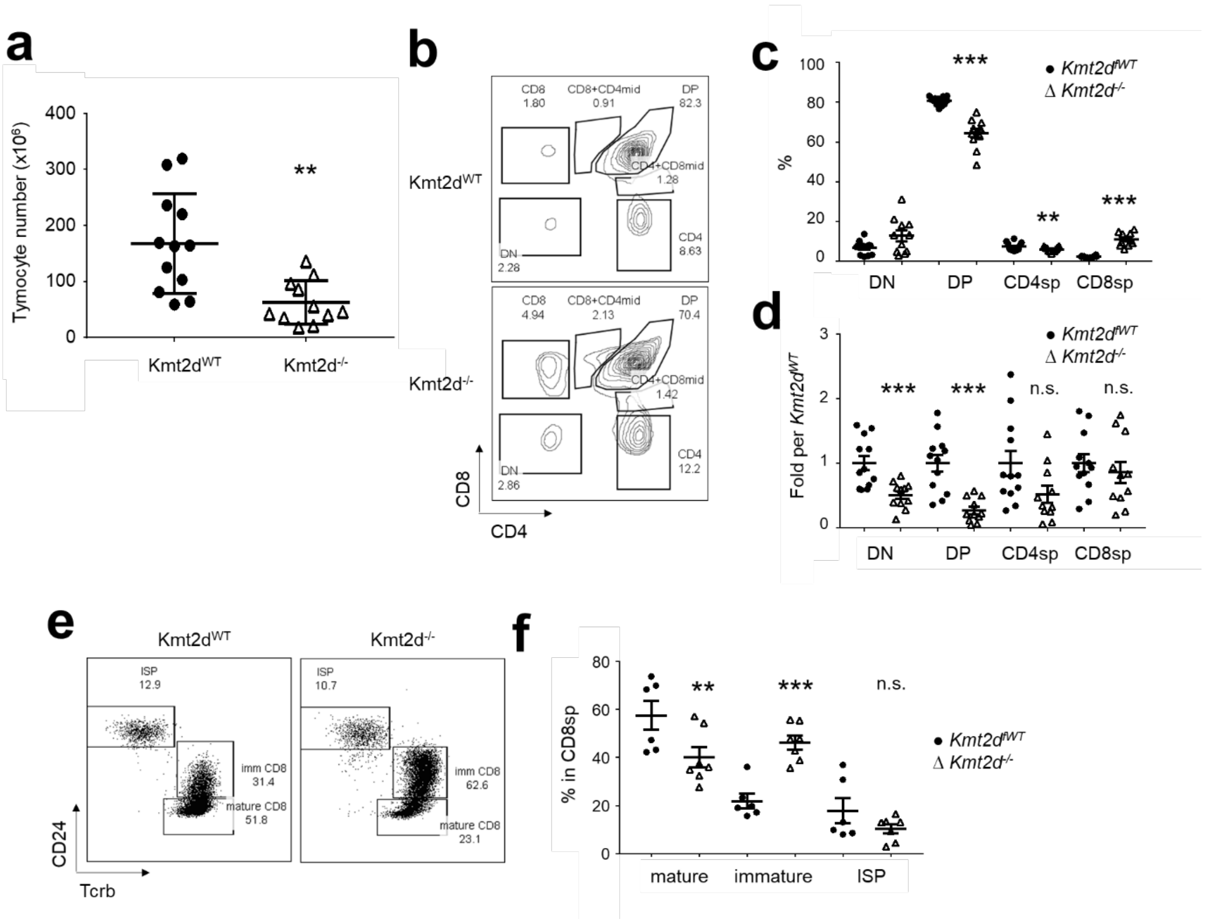

Supplementary Figure 2. Impaired thymic development with *Kmt2d* depletion. (a) Thymocyte count. (b-d) Thymic development recognized by CD4 and CD8 expression. Proportion in total thymocyte (c) and fold difference in absolute cell number (d). (e) Representative plot of $CD8^+$ SP cell maturation defined by Tcrb and CD24 expression. (f) Summary of $CD8^+$SP cell maturation. *, $p<0.05$; **, $p<0.01$; ***, $p<0.001$ based on Student's t-test.

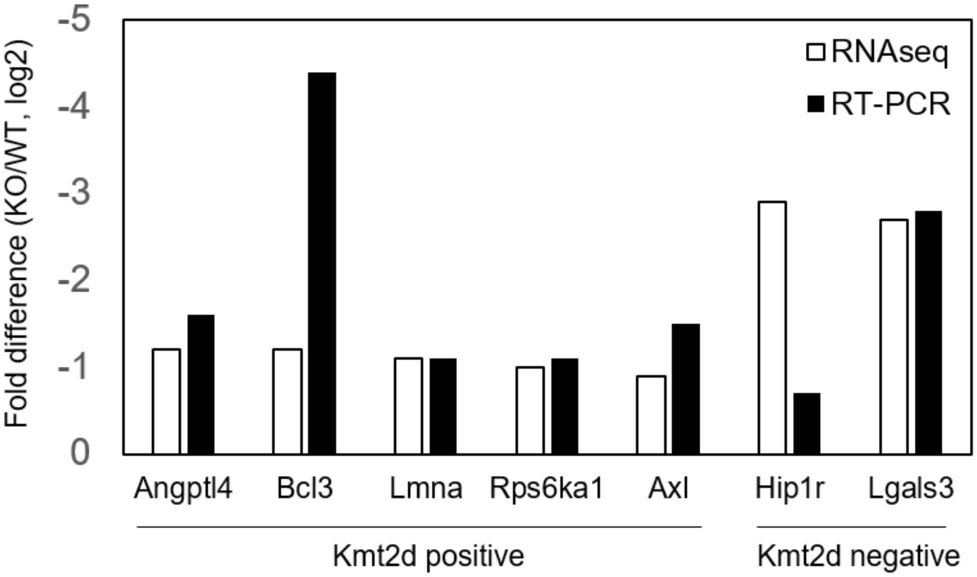

Supplementary Figure 3. Confirmation of reduced expression of apoptotic genes in Kmt2d KO mice. FACS-sorted naïve $CD8^+$ T cells from spleen of Kmt2d KO and Kmt2d WT mice were stimulated with immobilized anti-CD3 (3μg/ml) and soluble anti-CD28 (1μg/ml) antibodies for 24 hours. Quantitative RT-PCR analysis was carried out of seven genes and the gene expression ratios of KO/WT are presented. (N⩾3 for RT-PCR)
